# Patterns of biomedical science production in a sub-Saharan research center

**DOI:** 10.1186/1472-6939-13-3

**Published:** 2012-03-26

**Authors:** Selidji T Agnandji, Valerie Tsassa, Cornelia Conzelmann, Carsten Köhler, Hans-Jörg Ehni

**Affiliations:** 1 Medical Research Unit, Albert Schweitzer Hospital, Lambaréné, Gabon; 2 Institut für Tropenmedizin, Universität Tübingen, Tübingen, Germany; 3 Institut für Ethik und Geschichte der Medizin, Universität Tübingen, Tübingen, Germany

## Abstract

**Background:**

Research activities in sub-Saharan Africa may be limited to delegated tasks due to the strong control from Western collaborators, which could lead to scientific production of little value in terms of its impact on social and economic innovation in less developed areas. However, the current contexts of international biomedical research including the development of public-private partnerships and research institutions in Africa suggest that scientific activities are growing in sub-Saharan Africa. This study aims to describe the patterns of clinical research activities at a sub-Saharan biomedical research center.

**Methods:**

In-depth interviews were conducted with a core group of researchers at the Medical Research Unit of the Albert Schweitzer Hospital from June 2009 to February 2010 in Lambaréné, Gabon. Scientific activities running at the MRU as well as the implementation of ethical and regulatory standards were covered by the interview sessions.

**Results:**

The framework of clinical research includes transnational studies and research initiated locally. In transnational collaborations, a sub-Saharan research institution may be limited to producing confirmatory and late-stage data with little impact on economic and social innovation. However, ethical and regulatory guidelines are being implemented taking into consideration the local contexts. Similarly, the scientific content of studies designed by researchers at the MRU, if local needs are taken into account, may potentially contribute to a scientific production with long-term value on social and economic innovation in sub-Saharan Africa.

**Conclusion:**

Further research questions and methods in social sciences should comprehensively address the construction of scientific content with the social, economic and cultural contexts surrounding research activities.

## Background

Scientific production all over the world follows the pattern of core-periphery relationships in which the contribution of the periphery, including developing regions like sub-Saharan Africa, is marginal and depends on structure and infrastructure accessible through collaborative projects lead by institutions and scientists from core regions in Western Europe and North America. Thus, some authors assume that the practice of science in Africa would provide subsidiary sets of data to strengthen institutions, production and social value in the more developed world and would conversely generate research of only limited generalization and of little impact on the economic and social development of the local communities in peripheral regions [1-3].

So far, numerous studies addressed the questions related to the contribution of peripheral regions to worldwide scientific production. However, most of them use quantitative approaches as dominant methods targeting researchers in the frame of collaborative projects and assess internationally indexed databases. Overall, the results state that the works of African scientists registered in indexed databases are collaborative publications, but they pale in comparison to the rest of the world in terms of proportion and notoriety [4,5]. Additionally, a corpus of knowledge is generated with little potential for innovation and thus of poor social and economic value [5-7]. All these characteristics call an auxiliary type of scientific production to mind and may have prevented social scientists from focusing on scientific production in sub-Saharan Africa as a complex process with its own structures of centralization. However, numerous biomedical research institutions with a growing number of scientists are located in Africa. They collaborate with local, regional and international partners, which suggests that science is active in these areas. In this paper we describe the patterns of clinical research activities underlined by perceptions of scientists working in a "peripheral" research center in the sub-Saharan Africa region of Lambaréné, Gabon.

## Methods

### Study site

The first major stage in the development of the Medical Research Unit (MRU) of the Albert Schweitzer Hospital (HAS) lasted from its creation in 1981 to the end of the eighties. Since 1992, the site has succeeded in broadening its research activities to cover about 35,000 inhabitants living in the poor socio-economic conditions of the main town in Lambaréné and in the villages along the national road number 1 of Gabon.

Within a short period of time, a core group of researchers who live in Lambaréné and work permanently for the MRU has emerged. The core group of scientists interact with main collaborators from the German research institute and university as well as from numerous national, regional and international networks [8].

### Study participants

Members of the core research staff including investigators and field workers were asked to participate in the in-depth interview sessions. Interviews were conducted from June 2009 to February 2010. The following criteria were considered for the enrolment in the interview sessions. Research members who are experienced in the conduct of New Investigational Product Trials (NIP) and/or basic research, have regular interactions with research participants through study information, informed consent and/or clinical procedures and have a permanent position or intend to pursue a career as researcher or worker in the field of clinical research at the MRU were included in our study.

### The scope and the conduct of in-depth interviews

The scientific interests of the MRU focus on clinical trials in which novel drug combinations and vaccines against infectious diseases are being developed. Fundamental sciences such as molecular biology and immunology, which investigate the pathogenesis of infectious diseases are interest as well [8]. In order to succeed, the site has developed opportunities for international grant applications and is engaged in numerous public-private partnerships in which multicenter and transnational drug and vaccine trials are being conducted in Lambaréné. Moreover, the MRU has maintained close collaborations with institutions and scientists from Germany, the Netherlands and the United Kingdom. Therefore, it is likely that the MRU is dependent on the knowledge and abilities of scientists from wealthy core countries with regard to infrastructure and funding, suggesting that the patterns of core-periphery relationships may apply to the research being conducted at the MRU. The discussion about the abilities of MRU core researchers and the determinants that influence the conduct of clinical research in the social, economic and cultural contexts of Lambaréné was expected to support the understanding of how the scientific work is generated and perceived by the producers in a context of periphery dealing with core collaborators based in rich countries. Then, we postulated the interactions and relationships between scientists and research participants as essential in establishing science in the local contexts of Lambaréné and targeted the information procedures including the realization of informed consent and perception by scientists of the local communities' responses to research activities. The focus on informed consent as a transnational and relational procedure allowed us to discuss its production at transnational and local levels as well as ethical standards as part of the whole process of biomedical science production.

The interview sessions were semi open; the interviewees' speech was the focus, although the interviewer was able to direct on the main topics of the in-depth interview guide used to conduct the sessions. Interviews sessions were conducted in French, recorded, and transcribed for content analysis with regard to the interview guide.

## Results

### Characteristics of interviewees and patterns of clinical research

At the time the interview sessions took place, there were 26 people at the MRU whose job description was related to research including clinical, laboratory and field-based procedures. They were biologists, clinicians, nurses and field workers. Based on the administrative file of the MRU and the informal discussion with site directors, 12 fulfilled our main selection criteria (Table 1). Following the interviews analysis, scientific framework and local contexts surrounding clinical research activities were identified as the main patterns of the biomedical research at the MRU (Table 2)

**Table 1:** Characteristics of study participants

Study Identity	Duration at MRU (year)	Sex	Age	Position	Status	Interviews	Experience with research
INV1	5	M	33	Investigator	Middle	X*	PPP, AC
INV2	2	F	30	Investigator	Junior	X*	PPP, AC
INV3	10	M	55	Investigator	Senior	X*	PPP, AC
INV4	13	M	45	Investigator	Senior	X*	PPP, AC
INV5	2	F	30	Investigator	Junior	X*	PPP
INV6	7	M	60	Investigator	Senior	X*	PPP, AC
INV7	10	M	50	Investigator	Senior	X	PPP, AC
INV8	2	M	29	Investigator	Junior	X	PPP, AC
INV9	7	M	35	Investigator	Middle	X	PPP
FW1	13	M	35	Field Worker	Senior	X	PPP
FW2	8	F	35	Field Worker	Senior	X	PPP
FW3	3	M	25	Field Worker	Middle	X*	PPP

X* Quoted in the manuscript.

PPP: public-private partnerships sponsored project (transnational research)

AC: academic research for MRU initiated project

INV: investigator

FW: field worker

**Table 2:** Topics identified by content analysis and discussed in the manuscript

Framework of clinical research	Local contexts of clinical research
Transnational research	Characteristics of study participants in Lambaréné

Independent or academic research	

Ethical and regulatory guidelines and rules	The performativity of relationships and networks of research participants. Patient-physician relationships

	Patient-physician relationships

	Participant- investigator relationships

	Access to health care as "performative" tool for research purposes

### The framework of clinical research

#### Transnational research

Transnational studies are the most important part of research activities at the Medical Research Unit. The structure of typical transnational research conducted at the MRU includes partnerships and relationships between sponsor, research institutions, scientists in Europe and/or North America and the MRU and its scientists in technically and socio-economically poor conditions of a sub-Saharan Africa country. For instance, the MRU together with research centers in Benin Republic, Mozambique, and Kenya is evaluating antimalarial drugs as an intermittent preventive strategy against malaria in pregnant women on the field. The European partners are two research institutions that are coordinating scientific, technical and financial aspects of the project. The European Developing Countries Trials Partnership (EDCTP), a specialized funding organism of the European Union, funds the project.

In a similar framework of a malaria vaccine trial, a young investigator describes the scope of her work at the MRU: "*Our work on the field consists of vaccination, follow-up visits and also to ensure that the data collected are accurate and can be well analyzed and interpreted*".

Scientists of the MRU, however, may not be restricted to executing field procedures. They can contribute, for instance, to amending the study protocol and design, a more substantial scientific contribution that later impacts the field procedures, although any amendment should be done according to the rules defined by or with collaborators: "*Most of time, the sponsor comes with the study protocol already done. If there are scientific, medical or ethical concerns that are pointed out by us, we have to agree on changes to the protocol with the sponsor*". But, the process of protocol amendment seems very well "*structured, the study protocol is written in Europe, in the Netherlands for instance, for a multicenter study purpose. Modification and amendment to this protocol cannot be based on our willing without a green light from our collaborators*".

#### Academic or ancillary research

There is an opportunity for studies at the MRU that most of investigators call academic research. Academic research is either an investigator-based initiative of the MRU or an ancillary study to ongoing transnational research. Both are described as MRU's own research especially in terms of cognitive as well as operational contributions. The study idea, design, advocacy within the group, informed consent information, study conduct, data collection, analysis and publication are all carried out within the MRU group. Academic research is perceived as a source of scientific value and personal satisfaction as well as opportunity for career development, although investigators did not clearly state that transnational research is less valuable on an individual level. The links between transnational and academic research have been poorly discussed during our interviews; however, investigators did not claim the independence of their own studies over transnational projects. The study protocol design and the informed consent process are carried out with similar standards, guidelines and procedures for transnational and academic researches.

#### Guidelines and rules: Ethical and regulatory standards

The International Conference on Harmonization between the regulatory authorities of EU, Japan and USA with pharmaceutical companies has provided the main guidelines for conducting clinical trials around the world. The so-called Good Clinical Practice (ICH-GCP) have been referenced by the MRU's investigators as the main guidelines for performing their activities, although the guidelines of the Council of International Organizations for Medical Sciences (CIOMS) have also been mentioned as dedicated to ethical considerations specific to developing countries. GCP guidelines are considered as the document: "*that allows us to claim that we perform as elsewhere although environmental and cultural contexts may generate slight differences*"

The coherence between international standards and the national ethical and regulatory framework is perceived as an essential objective on the way to performing valuable science: "*The ICH-GCP has identified the stakeholders of research and their individual responsibilities*".

*"A similar approach should be implemented for our national regulations because research should follow the rules within a country"*. As noticed by a senior investigator, the other regions worldwide like *Europe "have a special bureau for the regulation of clinical trials activities and drug registration*". In Africa, biomedical research makes way for regulations which are however being performed according to the following claim of an interviewee: "*Local and international regulations should be harmonized to conduct research. Although it is a long journey, Gabon is working to build up ethical and regulatory systems for the management of biomedical research*".

The process of informed consent has been identified as the main procedure linking international requirements and local contexts for the purpose of conducting research of high standards.

"The informed consent process would be perceived as a means and strategy which allow a volunteer to be an active participant and to participate in the best way possible".

This view of a senior researcher at the MRU points to the challenges in applying ethical principles to the daily practice in a particular social and economic context. The process of informed consent is based on the widely shared idea to protect the freedom, safety and well-being of human participants during the experimental process: "*Over the last 15 years at the MRU, informed consent has been always obtained before any research onset, but now the compliance to the ethical committee requirements is better, although informed consent forms moved from 2 pages to around 10 pages*".

The requirements of ethical guidelines and regulatory authorities make some aspects of the informed consent form (ICF) difficult for both investigators and participants. A field worker and investigator have focused on a particular word that makes the process difficult. "Experimental" is a word that scares people and generates misconceptions. On the other hand, it is not possible to avoid this word because of ethical and regulatory requirements; it is perceived as an intention to hide the fact that research is an experiment and different from medical care. However, the requirement of informed consent should not necessarily be perceived as conflicting with views from the stakeholders involving: "*Regulatory and ethical sides are overseers; the most active are investigators and participants. Their interactions and relationships in the process would reflect the best possible, the need to have an informed person about the purposes of a proposed research as well as implications before participation*".

There is a variety of additional tools and strategies for researchers to deliver the best informed consent to their potential participants, including a community based team, public communication, and concise summaries of the ICFs-leaflets used for the purpose of informing and involving participants actively in the best possible way.

### Local contexts and clinical research: Research activities and their impact on community participation

#### Characteristics of research participants in Lambaréné

Freedom and willingness of members of the local community to participate in research have to emerge from scientific, social, economic and other determinants according to ethical principles as well as from the claims and attitudes of research stakeholders, including researchers at the MRU. A variety of determinants such as lack of education, low income, high disease burden, and limited access to health care has been identified as major factors that influence the community members to enroll in experimental procedures in Lambaréné by the interviewees. Social classes are a possible explanation for the "easiest enrolment" of rural people and economically disadvantaged city dwellers in research. In contrast, middle classes and educated people express more criticism of and reluctance towards research and even medical practices. However, our interviewees had conflicting opinions about the belonging to a particular social class of the research participants.

Social relationships and networks are either the main or the secondary reason for participating in research in Lambaréné according to our interviewees. In this relational system, participation is influenced by relatives, neighbours, the experience made by other research participants as well as the research team. Finally, many researchers have speculated on resources such as the type of communication, access and quality of health care and the role of the relationships and social networks as essential factors in characterizing the research participants in Lambaréné.

#### Relationships and social networks of research participants: Means to perform the social perception of clinical research

Attempts to summarize the characteristics of research participants and to emphasize the ethical concerns these characteristics generate may lead to the following assumption: in social and economic contexts of sub-Saharan Africa, like Lambaréné, both investigators and participants overstate the patient-physician relationship and enter into the experimental process with misconceptions and misuse of participant-investigator requirements. However, poverty, high burden of diseases, and the lack of or limited access to health care as essential drivers are not yet considered to be isolated motivations for individuals to participate in research. From the investigator's point of view, it is therefore essential to understand the value of a process such as that of informed consent procedure. Is it not just an inoperative process and merely a formality? For all interviewees, the social networks of research participants exist and may be impacted with variable resources such as human interactions, relationships, style of communication, type of study documentation, and access to health system. Relatives, neighbours, and other participants are also identified as important drivers for research participation, but interactions and relationships with the research teams, institution and participation in experimental procedures seem to be the more powerful tools that allow for the emergence of "informed, voluntary and less determined participants". Therefore, there is for most of our interviewees an advantage of conducting research in Lambaréné because the research purposes are shared and better understood than in other cities of Gabon, like "Libreville, Mouila", where no or limited research activities are carried out.

#### Patient-physician interactions and relationships in the process of participation in research

Availability and access to health care is an essential factor of conducting clinical research in Lambaréné. Health care has been perceived as the central resource and exchangeable good that supports a kind of deal between a participant in need and a researcher who wants to collect data for scientific purposes. Such a deal occurs during interactions in which each party has declared in written form that the entire process is based on the informed consent of the participant. Basically, if information is shared properly, the deal is acceptable. However, there are several factors that may make it harmful. First, the clinical researcher who collects data for scientific purposes is often a physician or nurse seen as caregiver by the participant. There is a continuum of roles and a difficulty to perceive the dividing line between research and routine care procedures for the participants. The burden of diseases and economic constraints influence this perception and the mix-up of research procedures with health care. The issues related to the "deal" have been identified as a major cause of poor-quality science due to the high rate of lack of compliance and withdrawal during the course of the study. On the other hand health-care provision during research on the participants has been perceived as a resource embedded in other resources that sustain the establishment of long term relationships between the research institution and the community. From this perspective, there is no exchange restricted to one item and limited in time. Access to highly qualified health care is promoted, and some researchers consider the research purposes as a continuum of that global health objective.

#### Participant-investigator relationships

The rate of people living in Lambaréné and vicinities with a positive view of the MRU and its research activities vary from 40% to 90% according to some interviewees who were asked to provide such estimation. Several reasons may support this broad range. For example, a wide variety of different studies are conducted at the MRU such as Phase I to Phase IV clinical trials, which assess the safety and efficacy of interventions, such as drugs and vaccines. Invasive procedures such as multiple blood sampling and bone marrow sampling are also performed.

The attitude towards research may also be influenced by the specific objectives and procedures of a study. Refusal rate in the same area for the same interventional trial differ significantly in two age categories. For example, parents are reluctant to enroll infants in trials, whereas they have fewer reservations about enrolling older kids. Participation and withdrawal of consent rates have different patterns in the same village for two different trials with different goals and procedures. In a vaccine trial, pressure on the research team to address safety concerns may vary significantly as the trial progresses. At the beginning, the rate of unexpected visits after drug or vaccine administration seems higher than during the course or at the end of the trial. All these cases where suggested by our interviewees as common features of the participants' endeavours related to study specific procedures.

## Discussion

This study reports the current patterns of clinical research activities including the framework structure and the contexts in which activities evolve in a sub-Saharan African center from the perspective of its key scientists and workers. Transnational research carried out through collaborations with mainly Western partners and locally initiated academic studies seem to be distinct types of research conducted at the MRU in Lambaréné, Gabon. In transnational collaborations, from the perspective of the core-periphery model, the scientists at the MRU may be considered as the "field workers" who collect data following the study protocol design and implementation by Western partners [9]. Additionally, the latter take care of the more complicated and demanding tasks such as managing the technical and financial side of the study and monitoring the study procedures to ensure the quality of the scientific production. Because Western partners provide outstanding scientific institutions and scientists as well as technology and financial resources, the main contents and models of scientific activities are elaborated predominantly by them. Thus, studies carried out in Africa may be late stage or confirmatory steps and not directly linked to local economic and social demands. For instance, applications of models elaborated by Western scientists and institutions on the biology and immunology of the malaria parasite in Lambaréné are likely to generate confirmatory data. Such research may be of limited value to the research center itself, which does not necessarily develop the ability to generate the models and is unlikely to stimulate innovation for economic and social development [6].

Objections may point to the above as a simplistic and partial interpretation of the perceptions of MRU researchers considering the development of clinical research in sub-Saharan Africa over the last decade. Beyond a rigid labour division of central versus peripheral activities, multinational trials can be considered as social organizations that involve human and non- human components with the ultimate goal of scientific production. From this perspective, there may be an opportunity for actors to impact both the natural course of relationships and the scientific contents and surrounding contexts of research activities.

As part of multinational research, international ethical and regulatory standards have been described by our interviewees as means to perform research as elsewhere, i.e. to requirements for generation of accurate and reliable scientific information. Even though there is similarity in scientific content, as it is set up in Western countries and can be applied in Africa, ethical and regulatory rules and processes are being dynamically implemented with regard to economic, social and cultural contexts with the ultimate goal of conducting high-quality research in Lambaréné. The local community's perception of research activities may be reduced to a less biased perception through daily interaction and consistent relationships with the research team, as attested by a possible emergence of participant-investigator relationships in our study setting and elsewhere [9-11].

Academic or ancillary studies are locally designed and conducted by MRU researchers. This type of research may be considered as an initiative to impact the course of the core-periphery relationships leading to scientific research and output that conform to the local long-term economic and social demands. Therefore, implementing international rules and guidelines while taking into account the local contexts as well as the scientific initiatives of local researchers may contribute to the emergence of biomedical institutions and scientists those activities may be more situated by the requirements and benefit of their local contexts. However, the production of science at the MRU has not been shown by researchers to be a dichotomous process of transnational versus local academic research. On the contrary, locally initiated studies are sometimes ancillary to transnational projects. This suggests that although transnational research may potentially reproduce the core-periphery model, it can also sustain the production and emergence of institutions and scientists in a peripheral region, like sub-Saharan Africa, with their own structure of centralization.

The recent development of public-private partnerships for research in sub-Saharan Africa in the context of millennium challenges and the existence of several biomedical research groups in sub-Saharan Africa countries is a new historical context. Here, we are reporting the patterns of research activities in a particular sub-Saharan research institution, namely the Medical Research Unit of Albert Schweitzer Hospital located in Lambaréné, Gabon. From the perspective of the MRU's researchers, transnational collaboration with outstanding Western institutions and scientists could limit the sub-Saharan science to subsidiary and confirmatory activities. However, this transnational collaboration, which implies scientific contents and ethical and regulatory rules, allows sub-Saharan scientists to perform more dynamically situated research and simultaneously impact the local community, its perception of research activities as well as the scientific contents with the potential of positively impacting the economic and social development in the future.

Limiting empirical material to the perceptions of the core researchers and workers at the MRU may be considered a weakness of this paper, seeing as though a single and unique conclusion and statement would be difficult. However, this study has made an important contribution to illuminating the complex and deep patterns of scientific activities and social contexts in sub-Saharan Africa while also taking international contexts into consideration. Importantly, the generation of interesting future research questions is also an important outcome of this study. So far social sciences have mainly assessed the impact and benefit of global interventions on the health-care systems in sub-Saharan Africa and the quantitative patterns of biomedical publications of the African scientists. This study points out the need for additional questions concerning the contents of scientific production as well as their links and potential impact on the long-term economic and social development in sub-Saharan Africa. To do so, we assume the posture, based on our finding, which an integrative approach would focus on specific questions to understand the dynamics of building-up the research institutions, the changes, if any on the elaboration on scientific contents considering the surrounding local economic, social and cultural needs. The methodological implications of such a project would be similar to the scientific frame at the Kenya Medical Research Institute, which considers the ethics of biomedical research in the contexts of sub-Saharan Africa and transnational research as a central process for the acceptability and validity of the global scientific enterprise in poor communities. More social science studies, research teams, and projects located within the clinical research site learning from the daily interactions with the scientists when they doing science [10-12] are needed. Additionally, as suggested by our findings, social and human activities as well as organizations are embedded, which allows one to assume that they should be studied using the paradigm that they affect each other in a dynamic manner [13,14].

## Conclusion

Future research questions including, but not limited to, interactions between transnational and locally designed studies, the impact of ethical and regulatory processes on the contents and the quality of the data generated, emergence of biomedical research structures like MRU and their networks through the study of individual scientists trajectories, the emergence of research participants and the interrelationships of biomedical research and public health would be essential for understanding the contribution of sub-Saharan African regions to scientific production.

## Competing interests

The authors declare that they have no competing interests.

## Authors' contributions

AST, CK and HJE have contributed for design of the study. AST, VT and CC have collected the study materials. AST has drafted the manuscript. All authors have contributed for the critical review of the manuscript.

## Pre-publication history

The pre-publication history for this paper can be accessed here:

http://www.biomedcentral.com/1472-6939/13/3/prepub
